# Automated Quantitative Extraction and Analysis of 4D flow Patterns in the Ascending Aorta: An intraindividual comparison at 1.5 T and 3 T

**DOI:** 10.1038/s41598-020-59826-2

**Published:** 2020-02-19

**Authors:** Sebastian Ebel, Josefin Dufke, Benjamin Köhler, Bernhard Preim, Benjamin Behrendt, Boris Riekena, Bernd Jung, Christian Stehning, Siegfried Kropf, Matthias Grothoff, Matthias Gutberlet

**Affiliations:** 1 Department of Diagnostic and Interventional Radiology, University of Leipzig – Heart Centre, Leipzig, Germany; 2 Department of Diagnostic and Interventional Radiology, University of Leipzig, Leipzig, Germany; 3 Department of Simulation and Graphics, University of Magdeburg, Magdeburg, Germany; 4 Department of Diagnostic, Interventional and paediatric Radiology, University of Bern, Bern, Switzerland; 5 Philips Clinical Science, Amsterdam, Netherlands; 6 Institute for Biometrics and Medical Informatics, University of Magdeburg, Magdeburg, Germany

**Keywords:** Aneurysm, Aortic diseases, Cardiovascular genetics

## Abstract

4D flow MRI enables quantitative assessment of helical flow. Current methods are susceptible to noise. To evaluate helical flow patterns in healthy volunteers and patients with bicuspid aortic valves (BAV) at 1.5 T and 3 T using pressure-based helix-extraction in 4D flow MRI. Two intraindividual 4D flow MRI examinations were performed at 1.5 T and 3 T in ten healthy volunteers (5 females, 32 ± 3 years) and 2 patients with BAV using different acceleration techniques (kt-GRAPPA and centra). Several new quantitative parameters for the evaluation of volumes [ml], lengths [mm] as well as temporal parameters [ms] of helical flow were introduced and analyzed using the software tool Bloodline. We found good correlations between measurements in volunteers at 1.5 T and 3 T regarding helical flow volumes (R = 0.98) and temporal existence (R = 0.99) of helices in the ascending aorta. Furthermore, we found significantly larger (11.7 vs. 77.6 ml) and longer lasting (317 vs. 769 ms) helices in patients with BAV than in volunteers. The assessed parameters do not depend on the magnetic field strength used for the acquisition. The technique of pressure-based extraction of 4D flow MRI pattern is suitable for differentiation of normal and pathological flow.

## Introduction

Time-resolved, 3-directional, 3-dimensional (3D) phase-contrast cardiac magnetic resonance (CMR) imaging (4D flow MRI) is an innovative technique for the assessment of cardiovascular disease. Beside absolute flow quantification, 4D flow MRI allows measurements of helical blood flow and may therefore provide deeper insights into cardiovascular pathologies^1^. Visual quantitative analysis of helical flow patterns can be performed already with appropriate reproducibility^2^. However, for more complex analyses, follow-up examinations or the assessment of therapeutic success, absolute quantification of helical flow patterns, e.g. of volumes or velocities within a helix, might be beneficial.

Numerous new parameters for the assessment of cardiovascular diseases with 4D flow MRI were already defined^3–6^ and different techniques for the semi-automatic assessment of helical flow have been described: recent studies utilized the λ_2_ criterion, vorticity, helicity or normalized helicity for evaluation of complex flow patterns in the ascending aorta^7–10^. However, it has been shown that most of those parameters are susceptible to noise and highly depend on sufficient image quality^11^. Therefore, we recently introduced a more stable and precise method: a pressure-based technique for helix extraction^12^, which is less susceptible to noise and will be used in this study.

The aim of our study was to utilize 4D flow MRI for the quantitative, pressure-based extraction and analysis of flow patterns in the ascending aorta in healthy volunteers and two patients with bicuspid aortic valves (BAV) as a proof of concept with different fast sequences at 1.5 T and 3 T.

## Methods

### Study cohort

Ten healthy volunteers with no history of cardiovascular diseases (CVD) (5 females, 22–52 years, mean age 32 ± 3 years) and two patients with BAV (1 female, age 39 and 45 years, referred to as BAV_1 and BAV_2) were included in this prospective, comparative study. A local ethics board approved the study and written informed consent was obtained from all participants. All experiments were performed in accordance with relevant guidelines and regulations.

### Magnetic resonance (MR) image acquisition

For the volunteers, two intraindividual CMR examinations were performed at two different days within a six month time span (mean 99 ± 61 days). The first datasets were acquired at a 3 Tesla (T) scanner using a 16-channel surface coil in combination with a 12-element spine coil (Magnetom Verio Dot, Siemens Healthcare GmbH, Erlangen, Germany) using a kt-GRAPPA accelerated sequence: TE 1.8 ms; TR 32 ms. This sequence has been evaluated before^13,14^. The second examinations were performed at a 1.5 T scanner using a five-element phased-array surface coil (Achieva, Philips Healthcare, Best, Netherlands): TE 2.3 ms, TR 37.6 ms. 4D flow MRI data were acquired in a sagittal oblique 3D volume covering the whole thoracic aorta. The flow-encoded velocity (Venc) was adjusted to a maximum flow velocity of 150 cm/s along all three flow directions. Variable parameters like field of view (280 × 260 × 52), Flip Angle (5°) and encoded phases (25) were kept constant in both examinations. Patients underwent CMR at 1.5 T, only. Data acquisition was performed in line with recently published recommendations^15^.

### Cardiac magnetic resonance (CMR) data analysis

#### Vessel segmentation, Blood flow visualization and Pre-processing

All processing, segmentation and measurement steps were carried out using *Bloodline*, a custom-made software tool for guided analysis of 4D flow MRI datasets, which has been introduced before^16–18^ (Department of Simulation and Graphics, University of Magdeburg, Germany). A centerline was drawn through the whole ascending aorta semi-automatically, beginning at the level of the aortic root. Anatomically, the ascending aorta was defined as the volume of the aorta between the aortic valve and the origin of the brachiocephalic trunk.

Bloodline enables the analysis of defined regions of interest, e.g. the ascending aorta, and provides the volume of the segmented vessel in ml as well as the length of the centerline in mm. Aortic blood flow was visualized using time-resolved pathlines. We corrected for phase wraps, eddy currents and background noise, as previously described^4,19^.

#### Measurements and flow quantifications

All segmentations and measurements were carried out by two observers with >5 years and 2 years of experience in CMR. Readers were blinded for which datasets were from which participant. A measuring plane was positioned at the mid-ascending aorta for 4D flow MRI volume quantification (ml/cycle) and peak velocity (m/sec). The automatic identification of complex flow patterns used by the software *Bloodline* utilizes a recently described pressure-based technique^12^. Both helical and vortical flow patterns produce pressure low-points, thus local variations of pressure are indicative of a helix or vortex. The calculation of the relative pressure map is based on an iterative method introduced by Tyszka *et al*.^20^ with additional post-processing steps. The resulting pressure map is smoothed using one iteration of a *3* × *3* × *3* binomial filter, clamped to the 1–99% quantile range to remove outliers. Lastly, to ensure that the pressure map is still relative to the average pressure after filtering, all values are shifted by subtracting the mean of the filtered pressure values. In the resulting pressure map, areas with a relative pressure P < 0 mmHg are considered to be vortex regions and used as a filter mask for densely seeded pathlines in order to visually represent the helical or vortical flow. Therefore, the relative pressure of P < 0 mmHg was used as a threshold to define regions with helical flow versus regions with non-helical flow. As this technique does not require any user interaction beyond the vessel segmentation, it might be used in a standardized manner and yields highly reproducible results.

However, the differentiation between helical and vortical flow might be difficult, because there is a smooth transition between both phenomena. Often, neither a visual qualitative nor quantitative differentiation between both is possible, because definite cut-offs do not exist. Visually, helical flow describes a rotational, corkscrew-like or helical flow pattern with a usually antegrade forward motion, whereas vortical flow is defined as a flow pattern with a revolving flow volume on the spot around a common axis without any forward motion^21^.

Since we could not quantitatively discriminate between both similar flow patterns and the assumption that a real vortex without any motion component in the direction of flow does not exist, we will use the common term “helix” or “helical” for both helical and vortical flow.

We introduced several new quantitative parameters to better describe helical flow patterns:

1. **T**emporal **H**elical **E**xistence **(TH**_**Ex**_**)** to describe the duration of helical flow throughout the cardiac cycle. This parameter is calculated from the time-resolved relative pressure map by measuring over how many time steps helical flow (i.e. negative pressure) is present, regardless of the size or number of detected helices.
  **TH**_**Ex**_ can only produce a single value, thus it is a global parameter for each dataset. The extracted duration is the sum of the duration of all helical flow according to the pressure map during the cardiac cycle.
  The calculation of the pressure map takes into account the entirety of the segmented vessel and thus, only depends on the segmentation quality. Defining a specific region of interest, e.g. the aortic arch, is independent of the pressure map calculation
  It was given as an absolute value (_**abs**_**TH**_**Ex**_) in milliseconds (ms) and as a relative value (_**rel**_**TH**_**Ex**_) in percent (%) of the cardiac cycle.
2. Different parameters were used to asses the size of helices:

2.1 The maximum **H**elical **V**olume **(HV**_**max**_**)** as an absolute value in milliliters [ml] and the relative value in percent [%] of the volume of the ascending aorta at a definite time point of the cardiac cycle.

**Figure 1:**
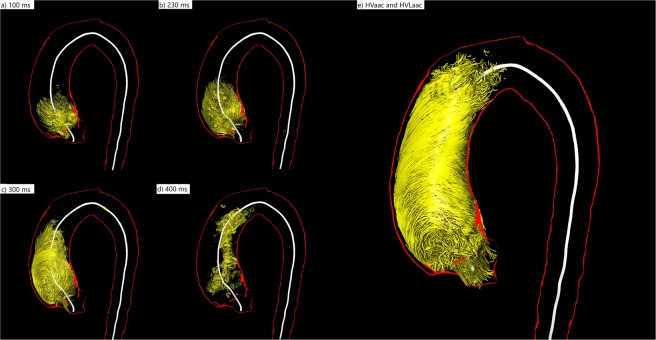
3D visualization of the evolution of a helix in the ascending aorta during the entire cardiac cycle of a patient with BAV (female, 39 years old) (**a**) At 100 ms the helix occupies only the very proximal part of the ascending aorta. (**b**) At 230 ms it has grown in volume and length and occupies almost half of the ascending aorta. (**c**) At 300 ms the very proximal part of the helix vanishes while the distal parts proceed towards the aortic arch. (**d**) At 400 ms only some remnants of the helix can be seen in the mid-ascending aorta. (**e**) The summation of all volumes that contributed to the helix during the entire cardiac cycle is the accumulated Helical Volume (HVacc). The absolute (relative) HVmax in this BAV patient was 44.81 ml (57.41%) of the ascending aorta volume. The absolute HVacc was 118.44 ml, the relative HVacc 92.44% of the ascending aorta volume. The temporal helical existence (THex) was 89% of the cardiac cycle, indicating that it persists almost throughout the entire systole and diastole. The absolute HVLacc in this patient was 107.8 mm, the relative HVLacc 100% of the ascending aorta length. HV(I)max was 22.32 ml/m^2^. HV(I)acc was 89.04 ml/m^2^.

2.2 Furthermore, the accumulated **H**elical **V**olume (**HV**_**acc**_) of the helix was defined as a summation of all volumes that contributed to the helix during the cardiac cycle. This parameter can be described as a temporal maximum intensity projection of the helix. It is given as an absolute value in milliliters [ml] or relatively in percent [%] based on the volume of the ascending aorta (see Fig. 1).

2.3 According to the **HV**_**acc**_ we assessed the accumulated length of a helix with the accumulated **H**elical Volume **L**ength (**HVL**_**acc**_). This parameter describes the length of a temporal maximum intensity projection of the helix. It is given as an absolute value in millimeters [mm] or relatively in percent [%] based on the length of the ascending aorta (see Fig. 1).

In addition, all volumetric parameters were given as an index in ml/m^2^, which describes the volume of the helix normalized to the body surface area (BSA in m^2^) as **HV(I)**_**max**_ and **HV(I)**_**acc**_.

The rotation direction (**RD**) was subdivided into left-handed and right-handed. If a helix rotates in flow direction clockwise around the centerline, it is considered as right-handed (**RD+**), otherwise left-handed (**RD−**). RD was assessed as described by Meuschke *et al*.^22^.

For all pathline segments, each consisting of a point and its successor, the RD is calculated separately. Each position of the line is projected onto the vessel cross-section plane at the nearest centerline point. This plane is defined by its center *c* (the centerline point) and two axes (x and y). The x-axis *nx* is determined by using the cross-product of the centerline tangent *n* at *c* and the word-space x-axis *1,0,0 T*.

**Figure 2:**
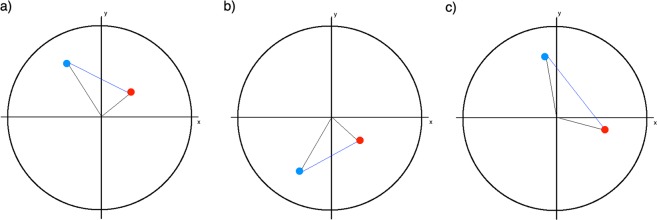
Three examples for the determination of the rotation direction in the cross-section. The lines from the blue point (first) to the red point (second) are pathline segments that are projected into a plane. (**a**) The successive points are in the first and second quadrant, they represent a right-handed segment. (**b**) The successive points are in the fourth and third segment, they represent a left-handed segment. (**c**) The points are in diagonal quadrants, in this case they represent a right-handed segment.

Similarly, the y-axis *ny* is the cross-product of *nx* and *n*. For the projected points, we identified the angle to the x-axis of the plane. For example, if the successive points are located in the first and second quadrant, they present an RD^+^ segment (Fig. 2a). If the successive points are in the third and fourth quadrant, a left-handed or RD− segment exists (Fig. 2b). If they are located in diagonal quadrants, the intersection between the connecting line and the y-axis is used to define a RD (Fig. 2c). Depending on whether a path line has more right or left rotating segments, it is classified as RD+ or RD−.

### Intra- and interobserver variability

The intraobserver variablility was assessed by one investigator with >5 years of experience in post-processing. This observer performed the previously described segmentations and measurements in all 12 datasets and repeated them in random order two months after the first evaluation. The interobserver variablility was analyzed by the second investigator, who repeated all segmentation and measurements as described above. The segmentation and measurements were done twice.

### Statistical analysis

All analyses were performed using MedCalc Statistical Software V15.11.4 (MedCalc Software, Ostend, Belgium). Quantitative variables were expressed as mean values and standard deviations (SD). A Wilcoxon test for comparison of temporal helical existence **(TH**_**Ex**_**)**, accumulated helical volume length (**HVL**_**acc**_), helical volume (**HV**_**max**_**, HV**_**acc**_) of volunteers at 1.5 T and 3 T was performed. A p-value < 0.05 was considered statistically significant. Additionally, correlation analyses were performed using paired correlations. Bland-Altman analysis provided the mean differences between measurements (bias) and the limits of agreement (LOA) used for the different methods of flow analysis. Intra- and interobserver variability was assessed using linear regression analysis, scatter plots and interclass correlation (ICC).

### Ethics approval and consent to participate

A local ethics board approved this study: Ethik-Kommission an der Medizinischen Fakultät der Universität Leipzig AZ 443/16-ek.

## Results

### Volunteer characteristics

Ten healthy volunteers and two patients with BAV with an equal gender distribution and age were chosen for this comparative study. The volunteer’s mean resting heart rate during the examination at 1.5 T and at 3 T did not differ significantly (p = 0.198) with 61/min ± 19 (1.5 T) and 60/min ± 18 (3 T), respectively, as well as the mean cardiac output (p = 0.303) during the two different time points with 4.47 l/min ± 1.45 (1.5 T) and 4.28 l/min ± 1.39 (3 T). In patients, the resting heart rate was 63 (BAV_1) and 67/min (BAV_2) and the cardiac output was 4.0 (BAV_1) and 4.8 l/min (BAV_2). The mean maximum diameter and volume of the ascending aorta was 30 mm ± 5 and 53.27 ml ± 15.91 in volunteers. The maximum diameter and volume of the aorta ascendens in patients were 33 mm and 71.91 ml (BAV_1) and 57 mm and 290.02 ml (BAV_2). The mean length of the ascending aorta was 92.4 mm ± 14.6 in volunteers and 88.3 (BAV_1) and 139.6 mm (BAV_2) in patients.

### CMR acquisition

Mean acquisition time was 8:09 ± 2:28 min at 1.5 T and 9:40 ± 3:15 at 3 T.

### Flow quantification

We found no significant differences between 3 T and 1.5 T regarding net forward flow [ml/cycle] and peak velocity [m/sec] in volunteers (see Table 1).

**Table 1:** Distribution of measurements of mean net flow and mean peak velocity in healthy volunteers at 1.5 T and 3 T.

Parameter	1.5 T	3 T	p-value	Correlation coefficient
Mean net forward flow **[ml/cycle] 1**^**st**^**/3rd quartile**	87.41 *81.11/94.18*	88.14 *81.54/94.86*	0.782	0.98
Mean peak velocity **[m/s] 1st***/***3rd quartile**	1.25 *1.09/1.49*	1.21 *1.08/1.36*	0.678	0.99

Distribution of measurements (including first and third quartile) of mean net flow and mean peak velocity in volunteers at 1.5 T and 3 T demonstrating no significant differences.

### Presence of helices in the ascending aorta

**Figure 3:**
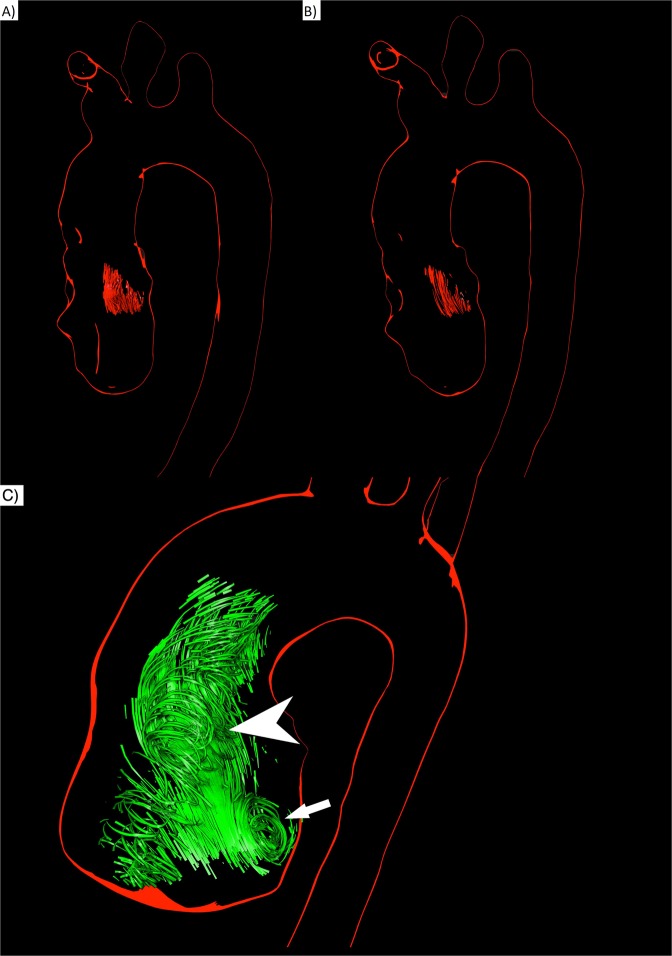
Comparison of 3D visualizations of helices in a healthy volunteer (male, 31 years old) at 1.5 T (**A**) and 3 T (**B**) HVmax 10.3 ml (**A**) vs. 11.0 ml (**B**); HVacc 16.9 ml (**A**) vs. 17.4 ml (**B**) (**C**) 3D visualizations of systolic helical and vortical flow in the ascending aorta of a patient with BAV (45 years old male) at 1.5 T. A large formation of vortical flow is located behind the opened aortic valve at the site of the aortic sinus (arrow). A formation of helical flow evolves through the ascending aorta (arrow head).

In all healthy participants helical flow could be detected only in the ascending aorta, at the same location at 1.5 T and 3 T. The automated helical flow detection of the software tool *Bloodline* detected one helix within the ascending aorta in every subject. The intraindividual comparison between helix visualizations at 1.5 T compared to 3 T in the same volunteer showed no significant differences (Fig. 3A,B). Visually, a clear corkscrew-like flow pattern could be observed. In every case the helix started close downstream from the aortic valve and evolved through the ascending aorta towards the aortic arch. The flow pattern at the very proximal parts of the observed helix could be further characterized as vortical flow in every participant, always located in the region of the coronary sinuses (Fig. 3C). Vortices were larger in patients than in volunteers. This was the only spot where vortical flow in the narrow sense occurred. The other parts of the helix could be classified as pure helical flow.

### Duration of helical flow in the ascending aorta throughout the cardiac cycle – the temporal helical existence (TH_Ex_)

**Figure 4:**
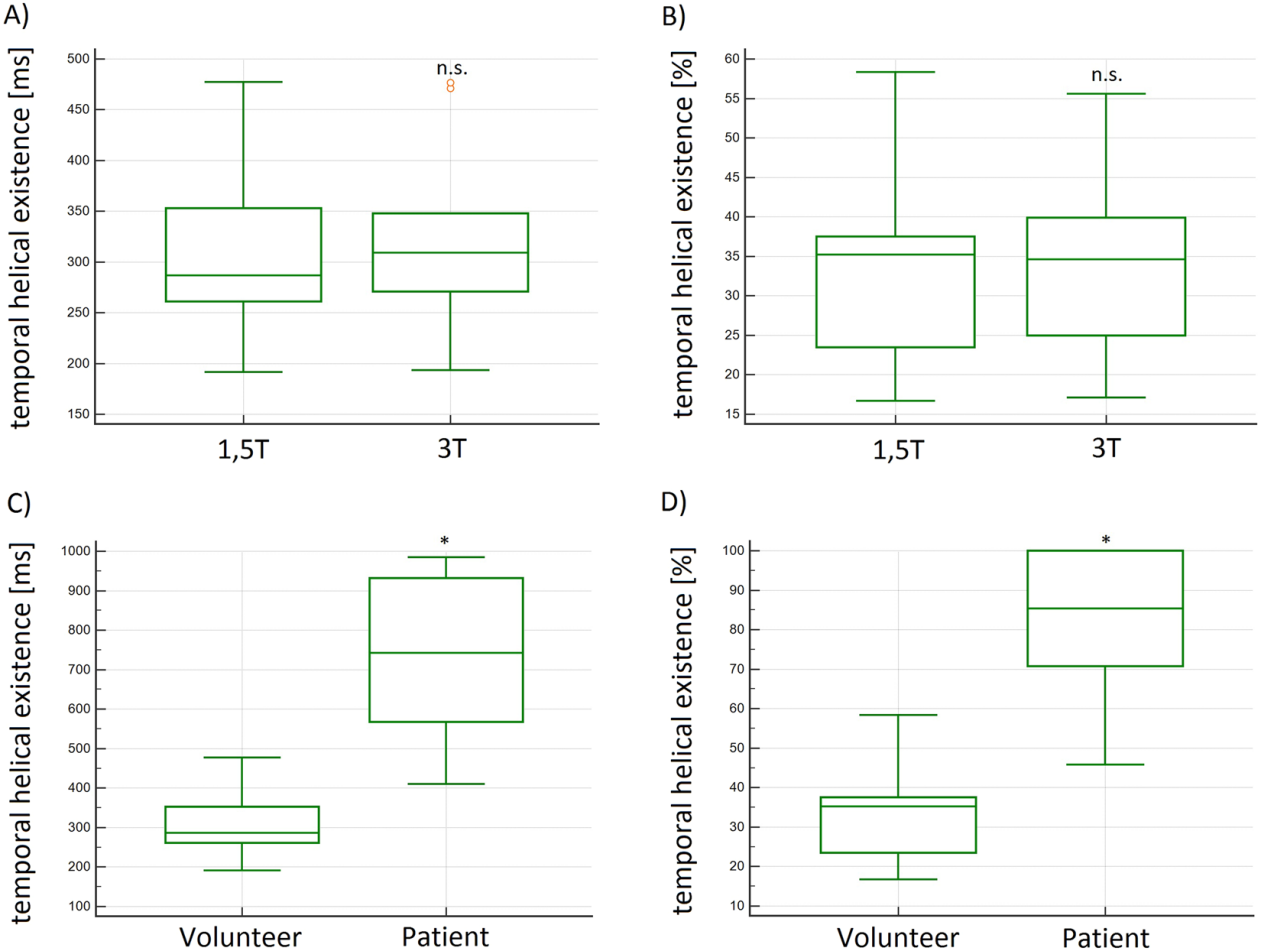
(**A**,**B**) Box plots of the absolute and relative temporal helical existence (TH_Ex_) in [ms] and [%] of all volunteers demonstrated no significant differences at 1.5 T and 3 T. (**C**,**D)** Box plots of the absolute and relative temporal helical existence (TH_Ex_) in [ms] and [%] demonstrated significantly higher values in patients as compared to volunteers. n.s. = no significant differences; *significant differences.

In volunteers the mean **TH**_**Ex**_ of the helices was 316.8 ms (±95.6) at 1.5 T and 320.7 ms (±92.8) at 3 T, respectively, without significant differences between the measurements (p = 0.448) (Fig. 4A,B). The correlation coefficient (R) was 0.99 and limits of agreement (LOA) were −31.1–21.2 ms (see Table 2). The helical flow started in early systole and faded away in the mid or late systole in all ten volunteers, independently from field strength. The **TH**_**Ex**_ in BAV_1 was 769.0 and 567.4 ms in BAV_2 and thus longer than in volunteers. Volunteer’s mean relative **TH**_**Ex**_ was 33.6% (±11.9) at 1.5 T and 34.0% (±11) at 3 T of the cardiac cycle, without significant differences between the measurements (p = 0.401, R = 0.99, LOA −4.1–3.5%) (see Table 2). The relative **TH**_**Ex**_ in patients was 100% in both cases and therefore more than 2-fold longer as compared to volunteers. In other words, in BAV patients helical lasted the whole cardiac cycle (see Fig. 4C,D).

**Table 2:** Summary of Mean Values, Standard Deviations (SD), and Bland-Altman analyses of the comparisons of volunteer examinations in 1.5 T and 3 T.

Parameter [Unit]	Mean (SD) 1.5 T	Mean (SD) 3.0 T	Correlation Coefficient R	p-value	Mean LOA [Unit]	Limits of agreement (LOA)
absolute temporal helical existence (**TH**_**ex**_) [ms]	**316.8 ms** (±95.6)	**320.7 ms** (±92.8)	0.99	0.437	−4 ms	−31.1–21.2 ms
relative temporal helical existence (**TH**_**ex**_) [%]	**33.6%** (±11.9)	**34%** (±11)	0.99	0.528	−0.4%	−4.1–3.5%
absolute maximum helical volume (**HV**_**max**_) [ml]	**11.7 ml** (±7.5)	**12.3 ml** (±7.1)	0.98	0.303	−0.5 ml	−2.4–3.3 ml
maximum helical volume index (**HVI**_**max**_) [ml/m^2^]	**9.9 ml/m**^**2**^ (±3.8)	**9.8 ml/m**^**2**^ (±3.8)	0.99	0.666	0.08 ml/m²	−0.9–1.1 ml/m²
relative maximum helical volume (**HV**_**max**_) [%]	**23.8%** (±17.4)	**24.9%** (±16.4)	0.98	0.325	−1.1%	−5.2–7.1%
absolute accumulated helical volume (**HV**_**acc**_) [ml]	**19.9 ml** (±9.0)	**19.7 ml** (±8.9)	0.99	0.640	0.2 ml	1.8–2.2 ml
relative accumulated helical volume (**HV**_**acc**_) [%]	**35.5%** (±19.9)	**37.9%** (±15.8)	0.99	0.339	−2.4%	−15.9–12.1%
absolute accumulated helical volume length (**HVL**_**acc**_) [mm]	**72.5 mm** (±11.8)	**73.0 mm** (±11.1)	0.99	0.417	−0.5 mm	−3.1–4.0 mm
relative accumulated helical volume length (**HVL**_**acc**_)[%]	**78.8%** (±13.7)	**79.3%** (±12.9)	0.99	0.406	−0.6%	−3.2–4.1%

Summary of the results of the calculated quantitative helical flow parameters in volunteer examinations at 1.5 T and 3 T demonstrating no significant differences.

### Maximum helical (HV_max_) and accumulated helical (HV_acc_) volumes in the ascending aorta

**Figure 5:**
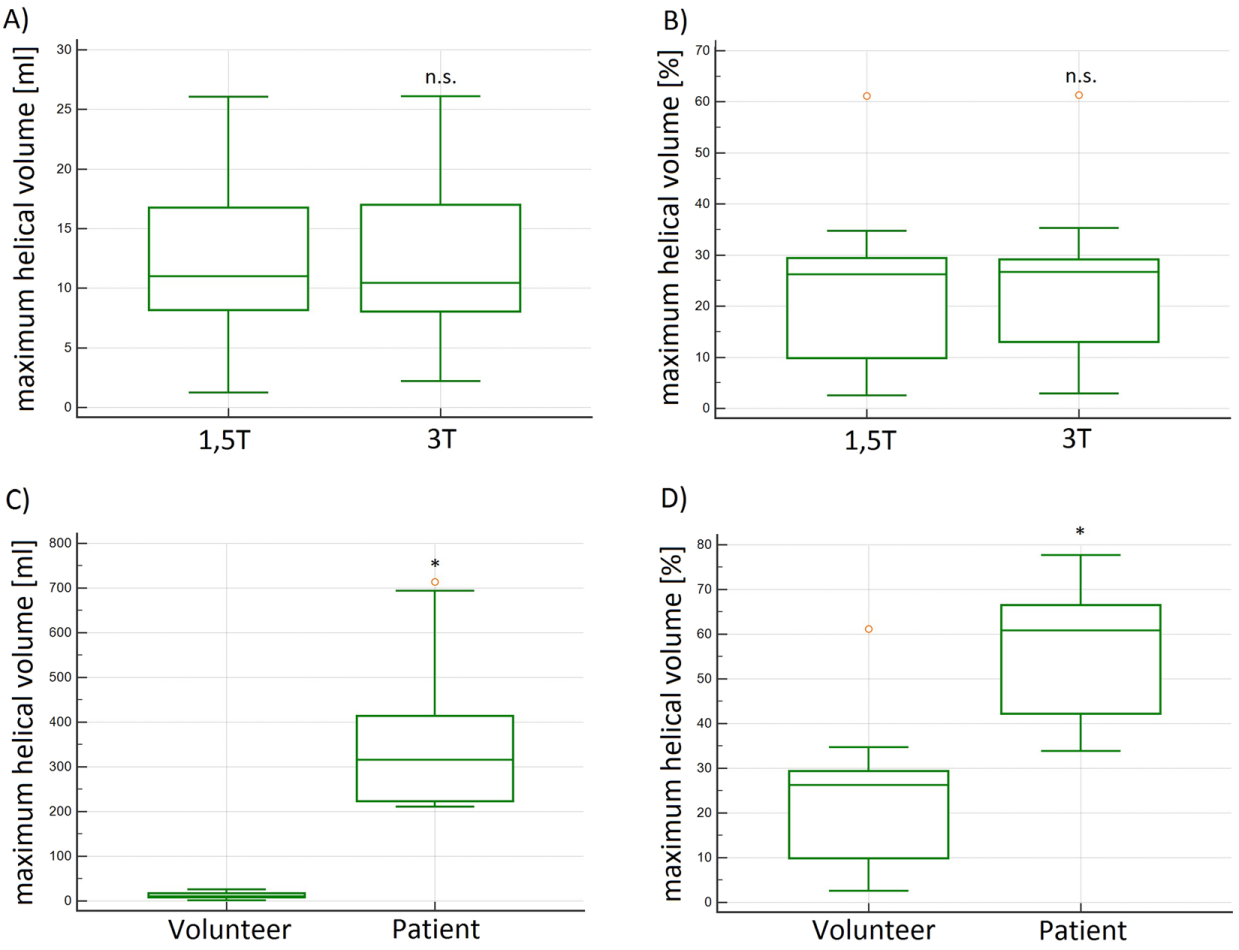
(**A**,**B**) Box plots of the absolute and relative maximum helical volume (HV_max_) in [ml] and [%] demonstrated no significant differences at 1.5 T and 3 T in volunteers. (**C**,**D**) Box plots of the absolute and relative maximum helical volume (HV_max_) in [ml] and [%] demonstrated significantly higher values in patients as compared to volunteers. The difference of the relative HV_max_ is less pronounced as with the absolute values. n.s. = no significant differences; *significant differences.

The volunteers’ absolute and relative **HV**_**max**_ demonstrated a broad range of volumes from 2.9–19.5 ml (9.9–34.7%) at 1.5 T and 2.6–19.4 ml (10.0–35.2%) at 3 T. The mean absolute or relative **HV**_**max**_ was 11.7 ml (±7.5) or 23.8% (±17.4) at 1.5 T and 12.3 ml (±7.1) or 24.9% (±16.4) at 3 T. We found no significant differences between the measurements at 1.5 and 3 T (p = 0.628 and p = 0.640) (Fig. 5A,B). The correlation R was 0.98 and the LAO were −2.4–3.3 ml and −5.2–7.1%, respectively (see Table 2). The absolute **HV**_**max**_ in patients was larger with 41.8 ml (BAV_1) and 77.6 ml (BAV_2), but not the relative **HV**_**ma**x_ with 17.4% (BAV_1) and 32.3% (BAV_2) (Fig. 5C,D). In all cases the helices reached their maximum volume within the first 30% of the cardiac cycle.

**Figure 6:**
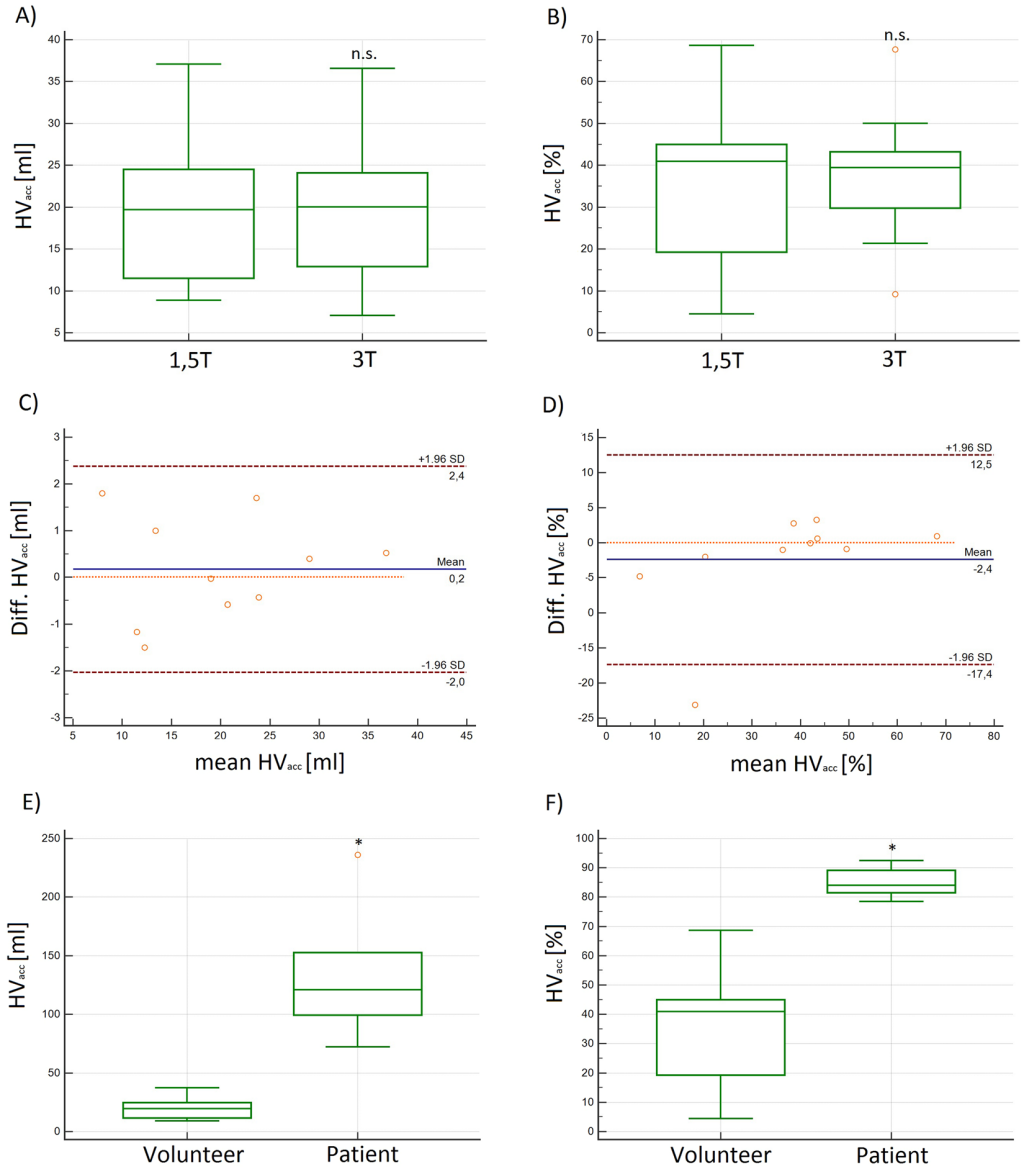
(**A**,**B**) Box plots and (**C**,**D**) Bland-Altman plots of the absolute and relative accumulated helical volume (HV_acc_) in [ml] and [%] at 1.5 T and 3 T demonstrating no significant differences in volunteers. **(E,F)** Box plots of the absolute and relative accumulated helical volume (HV_acc_) in [ml] and [%] in patients and volunteers. The difference of the relative accumulated HV_acc_ is less pronounced as with the absolute values, but still significant. n.s. = no significant differences; *significant differences.

The mean absolute or relative **HV**_**acc**_ in volunteers was 19.9 ± 9.0 ml or 35.5 ± 19.9% at 1.5 T and 19.7 ± 8.9 ml or 37.9 ± 15.8% at 3 T without significant differences between measurements (p = 0.501 and p = 0.417) (Fig. 6A,B). The correlation coefficient (R) was 0.99 and the LOA were −1.8–2.2 ml and −15.9–12.1% (see Table 2 and Fig. 6C,D)).

The mean absolute and relative **HV**_**acc**_ in patients was larger with 55.9 ml in BAV_1 and 236.1 ml in BAV_2 (77.7 and 81.1%) (Fig. 6E,F).

**Figure 7:**
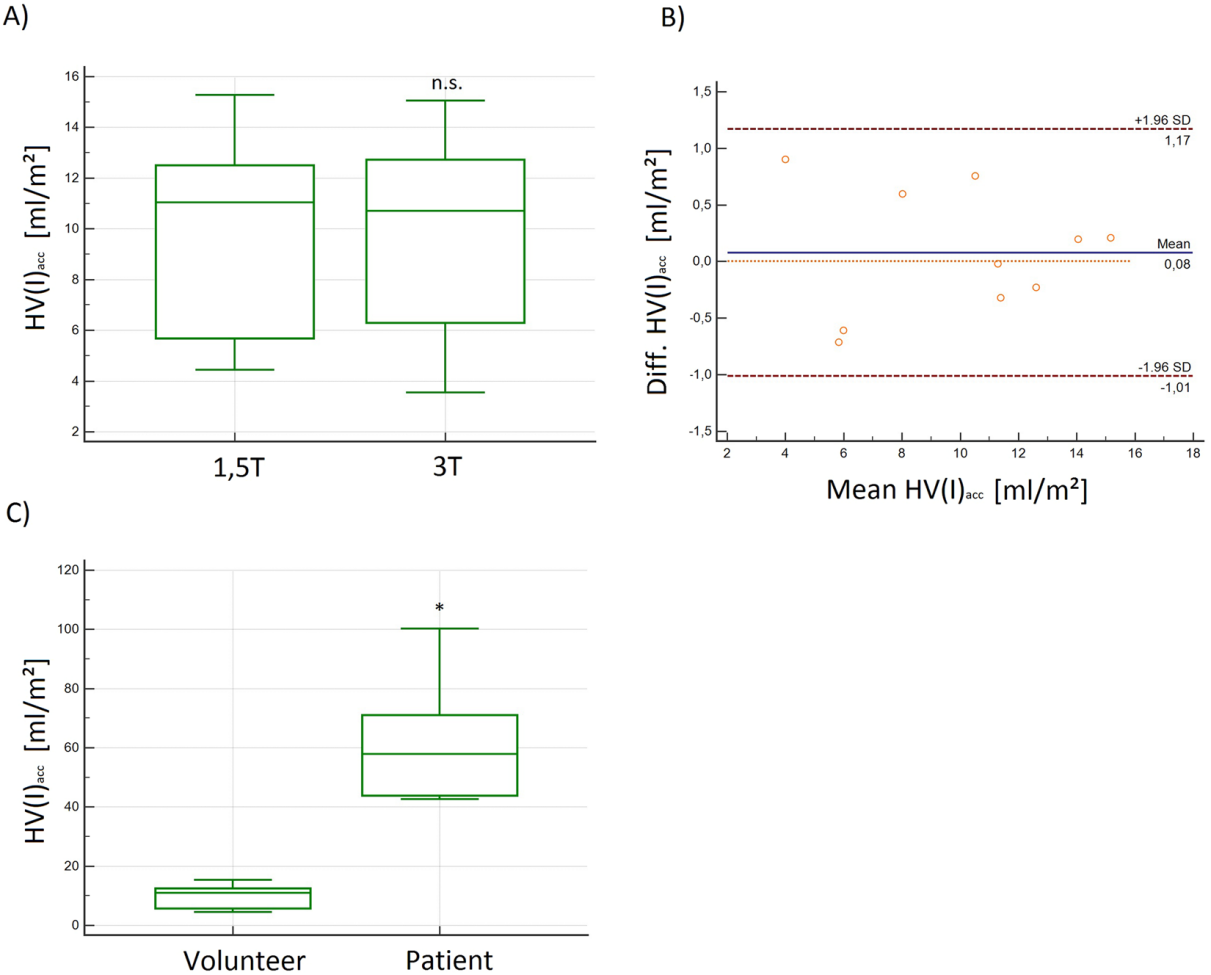
**(A)** Box plot and **(B)** Bland-Altman plot of the absolute and relative maximum Helical-Volume-Index (**HV(I)**_**acc**_) demonstrated no significant differences at 1.5 T and 3 T in volunteers. **(C)** Box plot of the absolute HV(I)_acc_ demonstrating significantly higher values in patients as compared to volunteers in [ml/m^2^]. n.s. = no significant differences; *significant differences.

Additionally, we introduced the accumulated and maximum helical volume index, (**HV(I)**_**acc**_ and **HV(I)**_**max**_), indicating the accumulated and maximum volume of a helix normalized to the BSA. With the introduction of these parameters we wanted to reduce the broad range of **HV**_**acc**_ and **HV**_**max**_, assuming that these parameters were highly depending on the shape and weight of each individual. The **HV(I)**_**acc**_ and **(HV(I)**_**ma**x_) ranged from 3.5 to 15.3 (0.6 to 13.6) ml in volunteers and up to 100.2 (72.2) ml/m^2^ in patients. The volunteers’ mean calculated **HV(I)**_**acc**_ was 9.9 ± 3.8 at 1.5 T and 9.8 ± 4.1 ml/m^2^ at 3 T without significant differences (p = 0.558), LOA were −1.0–1.2 (Fig. 7A,B). **HV(I)**_**max**_ was 6.1 ± 4.0 ml/m^2^ at 1.5 T and 6.2 ± 4.1 at 3 T without significant differences between the measurements (p = 0.631, R = 0.99). Compared to **HV**_**acc**_ and **HV**_**max**_ the SD of these indeces was lower. In patients the **HVI**_**acc**_ was 37.4 (BAV_1) and 100.2 ml/m^2^ (BAV_2) and the **HVI**_**max**_ in BAV_1 was 41.8 ml and in BAV_2 77.6 ml (Fig. 7C).

In conclusion, all absolute and relative temporal and volumetric helical parameters were larger in patients than in volunteers, except the relative maximum helical volume (**HV**_**max**_).

### Accumulated helical volume length (HVL_acc_) of helices in the ascending aorta

**Figure 8:**
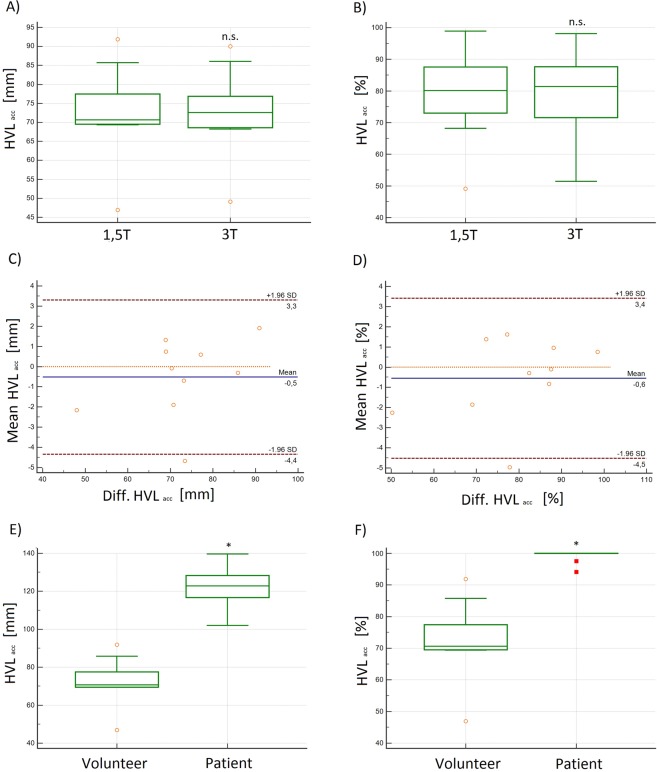
**(A,B):** Box plots and **(C,D)** Bland-Altman plots of the absolute and relative accumulated helical volume length (**HVL**_**acc**_) in [mm] and [%] demonstrated no significant differences at 1.5 T and 3 T in volunteers. **(E,F)** Box plots of the absolute and relative accumulated helical volume length (**HVL**_**acc**_) in [mm] and [%] demonstrating significantly higher values in patients as compared to volunteers. n.s. = no significant differences; *significant differences.

The volunteers’ mean absolute or relative **HVL**_**acc**_ was 73 ± 12 mm or 78.8 ± 13.7% at 1.5 T and 77 ± 11 mm or 79.3 ± 12.9% at 3 T without significant differences between the measurements (p = 0.411 and p = 0.491) (Fig. 8A,B). The correlation coefficient (R) was 0.99 and the LOA were −3–4 (absolute **HVL**_**acc**_ [mm]) and −3.2–4.1 (relative **HVL**_**acc**_ [%]) (see Table 2 and Fig. 8C,D).

The absolute (relative) **HVL**_**acc**_ in BV_1 was 88 mm and in BAV_2 140 mm (100%) (Fig. 8E,F). In all cases **HV L**_**acc**_ was larger in patients than in volunteers.

### Rotational direction (RD) of helices in the ascending aorta

In all cases helical flow was right-handed (RD+); we found no left-handed helical flow in healthy volunteers as well as in our two patients.

### Intra- and interobserver variability

Analysis of the intraobserver variability showed for the measurements of absolute **TH**_**Ex**_, **HV**_**acc**_, **HVL**_**acc**_ and **HV**_**max**_ R = 0.99, 0.99, 1.0 and 1.0 (p < 0.0001), ICC was 0.99, 1.0, 1.0 and 0.99, respectively.

Analysis of the interobserver variability showed for the measurements of absolute **TH**_**Ex**_, **HV**_**acc**_, **HVL**_**acc**_ and **HV**_**max**_ R = 0.96, 0.97, 0.98 and 0.99 (p < 0.0001), ICC was 0.97, 0.95, 0.98 and 0.97, respectively.

## Discussion

The software *Bloodline* utilizes a pressure-based technique for the assessment of flow patterns. The major finding of this study is the consistency of quantitative 4D flow MRI parameters for the assessment of helical flow across platforms and field strengths using new software for helix extraction. The results demonstrated that these parameters neither depend on the magnetic field strength nor on the scanner platform used for the acquisition. In addition, we demonstrated as a proof of concept that there are significant differences between healthy volunteers and patients with BAV.

For establishing normal values or for planning multi-center trials, scanner- and center-independent parameters are mandatory. If this prerequisite is not fulfilled, each center has to establish its own normal values. Therefore, the Society for Cardiovascular Magnetic Resonance (SCMR) recommends to establish normal values for every particular set-up (scanner, vendor, field strength, contrast regime, sequences)^23^ when using different T1- and T2-mapping techniques.

We performed 4D flow MRI measurements at different scanners using different sequences, acceleration techniques and different field strengths and found no significant differences. Thus, we state that those parameters could serve for establishing normal values or for follow-up examinations in a clinical setting with a possible multi-scanner application.

There are different methods for identifying flow patterns, which can be divided into local and global methods^24^. For example, the λ_2_ criterion by Jeong and Hussain and the Q criterion by Hunt are local methods^25–27^. Recent studies utilized vorticity, helicity and normalized helicity for assessment of complex flow patterns, which are local methods as well. In 2013 Köhler *et al*. found that those methods depend highly on sufficient image quality, otherwise they could misinterpret noisy regions for helices^11^. Compared to local methods, global methods give more precise results at the expense of higher computation times^11^. One example of a global method is a technique based on local pressure minima. Köhler *et al*.^12^ validated this technique by visual inspection of the helix regions and comparison with the commonly used and accepted λ_2_-based helix detection on 10 4D flow MRI datasets (1 healthy volunteer, 3 aortic aneurysms, 2 BAV, 1 aortic arch prosthesis, 1 aortic bypass, 1 left ventricle and atrium with valve defect, 1 insufficient pulmonary valve). In most cases, pathlines filtered using the pressure-based method produced a more satisfying visual representation of the helix compared to a λ_2_-based filtering, e.g. as a result of increased line density and less fragmented lines, whereas in all other cases the visual quality was at least on par. The analysis of the inter- and intraobserver variability showed that the introduced technique and parameters yield highly reproducible results.

Although there was a six month time span between the two volunteer examinations, we found no significant differences between the volunteer groups regarding net forward flow, peak velocity and helical flow volumes in the ascending aorta and cardiac output, which underlines the stability of measurements in 4D flow MRI across platforms and even with different magnetic field strength. These findings fit well with the literature: In 2012 Strecker *et al*. found identical performance of 4D flow MRI at 1.5 T and 3 T regarding flow velocities and volumes^28^.

Contrarily, we found significant differences between healthy volunteers and patients with BAV. More importantly, for all assessed parameters we found significantly higher results in patients than in volunteers, indicating that the used method of pressure-based analysis enables a distinction between normal and pathological flow. The technique of pressure-based helix analysis could therefore serve for detailed assessment of pathologies of the cardiovascular system. This should be addressed in further studies.

With the introduced technique of pressure-based helix extraction we were able to detect one helix in the ascending aorta in every participant. These findings partially match with the results of previous studies indicating that helical flow in the ascending aorta is a physiological phenomenon. Moreover, helical flow is thought to protect against atherosclerosis^8,29^. However, in 2011 Morbiducci *et al*. analyzed flow patterns in the thoracic aorta in five healthy volunteers and found no helical flow in the ascending aorta or the proximal aortic arch. These analyses have been carried out by visual pathline tracking only, with possible misinterpretation due to an overlay of the pathlines with helical flow and pathlines with non-helical flow. In contrast, by utilizing a technique enabling semiautomatic extraction, visualization and analysis of helical flow separately from the pathlines with non-helical flow, we found one helix in the ascending aorta close downstream from the aortic valve in each volunteer.

Recently, the evolution of helical flow has been investigated and it has been stated that helical flow emerges mainly in peak systole^8^. Contrarily, our results indicate that helical flow usually starts already in early-systole and ceases in late-systole in healthy volunteers or even last the whole cardiac cycle in BAV patients. Additionally, we found that helices in the early systole are rather small, while they reach their maximum volume and length around mid-systole in both patients and volunteers.

One possible explanation for those differences could be that in visual analyses small helices could be missed due to the overlay of pathlines with helical flow and pathlines with non-helical flow, while (semi)automatic analyses enable detection of even small helices.

In 2012 Bürk *et al*. e.g. assessed 3D blood flow patterns like the helical volume, but they evaluated the helical volume only visually. They discriminated whether a helix partially or fully filled the aortic lumen and found rather small helices in healthy participants^21^. These findings fit with our results: In the study presented here, we found a partial filling of the aortic lumen in all volunteers and a complete filling in patients. More importantly, we were able to absolutely quantify helical flow. Furthermore, we introduced additional parameters for the quantitative assessment of flow patterns in the ascending aorta like the absolute or relative accumulated Helical Volume (**HV**_**acc**_) and the accumulated Helical Volume Length (**HVL**_**acc**_).

We showed that these parameters could help to identify and precisely quantify helical flow. We found that the temporal existence (ms), volume (ml) and length (mm) as absolute values strongly depend on individual characteristics of the volunteers. Therefore, they might not be suitable for inter-individual comparisons. It seems to be reasonable to prefer normalized values like the helix’ temporal existence normalized to the duration of the cardiac cycle, its volume normalized to the volume of the ascending aorta or on the BSA and its length normalized to the length of the ascending aorta.

For the assessment of the dimensions of the right and left ventricle it is recommended to normalize the volumes of the ventricles to the subject’s BSA^30^. Analogous to this normalization we introduced the maximum Helical Volume index (HVI_max_), which represents the maximum helical volume of the helix normalized to the BSA. We found the HVI_max_ to be significantly higher in patients with BAV than in volunteers, indicating that this parameter could serve for the assessment of congenital malformations.

Additionally, we introduced the accumulated Helical Volume (**HV**_**acc**_) and Helical Volume Length (**HVL**_**acc**_) and found those parameters to be significantly higher in BAV patients compared to healthy volunteers.

It is known that somehow bigger and longer helices with a “mismatch” between the volume of the helix and aorta could lead to aortic dilatation or elevated wall shear stress^21,31,32^. However, further studies are needed to evaluate which parameters are best for the assessment of pathologic flow patterns.

In previous studies, three-dimensional helical parameters have mainly been assessed quantitatively only in several two-dimensional cross-sections distributed over the aorta^2,33^. These methods enabled e.g. precise measurements of the rotational direction of helices. However, since these measurements were performed in two-dimensional cross-sections of the aorta, they could only cover a small part of the helix and thus e.g. precise measurements of the duration, volume and length of a helix could not be performed. In our study, we introduced a comprehensive technique enabling 3D analysis of helices with full coverage.

Previous studies utilizing only a visual assessment of 4D flow MRI patterns in the aorta showed that vortical flow is a common phenomenon at the coronary sinuses and seems therefore to be a physiological phenomenon^34^. This is well in line with our findings: we semi-automatically detected vortical flow at the coronary sinuses and helical flow in the ascending aorta in every subject.

The main limitation of this study is that only 10 healthy individuals and two patients (one with a dilated aorta) were included. For further validation of the results a larger study cohort is needed, however, this study was designed as a proof of concept and an introduction of new parameters.

In conclusion, the technique of pressure-based extraction of 4D flow MRI pattern is suitable for quantitative analysis of helical flow in the ascending aorta and for differentiation of normal and pathological flow. It yields results that are independent from the used sequences, strengths of the magnetic field and manufacturers. With this technique, it might be possible to perform follow-up examinations and to quantitatively assess therapeutic success. These parameters could possibly give deeper insights and lead to a better understanding of arterial hemodynamic and pathology.

## Data Availability

Please contact author for data requests.
